# Immunological factors, important players in the development of asthma

**DOI:** 10.1186/s12865-024-00644-w

**Published:** 2024-07-26

**Authors:** Yang Wang, Li Liu

**Affiliations:** Department of Pediatric Respiratory, Children’s Medical Center，The First Hospital of Jilin University, Changchun, 130021 China

**Keywords:** Asthma, Imbalance, Immunologic, Monoclonal antibody

## Abstract

Asthma is a heterogeneous disease, and its development is the result of a combination of factors, including genetic factors, environmental factors, immune dysfunction and other factors. Its specific mechanism has not yet been fully investigated. With the improvement of disease models, research on the pathogenesis of asthma has made great progress. Immunological disorders play an important role in asthma. Previously, we thought that asthma was mainly caused by an imbalance between Th1 and Th2 immune responses, but this theory cannot fully explain the pathogenesis of asthma. Recent studies have shown that T-cell subsets such as Th1 cells, Th2 cells, Th17 cells, Tregs and their cytokines contribute to asthma through different mechanisms. For the purpose of the present study, asthma was classified into distinct phenotypes based on airway inflammatory cells, such as eosinophilic asthma, characterized by predominant eosinophil aggregates, and neutrophilic asthma, characterized by predominant neutrophil aggregates. This paper will examine the immune mechanisms underlying different types of asthma, and will utilize data from animal models and clinical studies targeting specific immune pathways to inform more precise treatments for this condition.

## Introduction

Asthma is a common respiratory ailment that affects more than 300 million people globally. This ailment manifests itself through chronic airway inflammation, airway hyperresponsiveness (AHR), reversible airflow limitation, etc. Notably, the syndrome manifests as the infiltration of diverse inflammatory cells, including eosinophils, neutrophils, and lymphocytes, and the involvement of inflammatory mediators and cytokines. Asthma has a significant impact on human health worldwide, and there is an urgent need to improve our understanding of its pathogenesis. The establishment of asthma phenotypes and endotypes provides potential for increased accuracy in disease classification and the exploration of suitable biomarkers and therapeutic targets [1–3]. T-cell subtypes, including Th1 cells, Th2 cells, Th17 cells, Tregs, and their corresponding cytokines, are significant contributors to the development of asthma. Drug therapy targeting immune molecules offers new therapeutic prospects for asthma patients [4–6].

## Imbalance in the immune response of Th1 and Th2 cells

T helper type 1 (Th1) cells and Th2 cells are important effector cells that inhibit each other during the development of asthma [7]. Th1 cells mainly mediate the cellular immune response, delay hypersensitivity and assist in the synthesis of phagocytosis-related antibodies, e.g., IgG, IgM and IgA, which may inhibit the development of asthma [8]. Th2 cells primarily mediate the humoral immune response and immediate hypersensitivity [9]. They also aid in the proliferation of B cells, IgE synthesis, antibody production and non-phagocytic host defense, contributing to the development of asthma. These cells are derived from the same group of precursor cells (Th0) and are induced by different cytokines, and the direction of T-cell polarization is closely related to the local cytokine environment in which dendritic cells (DCs) play a significant role. According to previous research, type I dendritic cells (DCIs) exhibit poor maturation and insufficient IL-12 secretion, which prevents naïve CD4 + T cells (Th0) from differentiating into Th1 cells [10]. However, when Th0 cells are stimulated with IL-4, DCII promotes the differentiation of Th0 cells into Th2 cells, which causes Th1 cells to secrete less IFN-γ and Th2 cells to secrete more IL-4 [11, 12]. Several additional factors have been shown to affect the selective development of Th1 and Th2 cells. These factors include the dosage and affinity of the antigen, major histocompatibility complex (MHC) haplotypes, and co-stimulatory factors. The Th1 cell-specific transcription factor T-bet binds to the Th2 cell-specific transcription factor GATA-3, hindering the binding of GATA-3 to Th2 cytokine genes and suppressing the production of Th2 cytokines, and vice versa. A decrease in the level of Th1 cells expressing T-bet in the airways of asthma patients caused an increase in GATA-3 expression and Th2 cytokine production. Increased GATA-3 inhibits Th1 cytokine production through the inhibition of STAT4, resulting in a further increase in respiratory Th2 cells in asthma patients [13].

For many years, an imbalance in Th1/Th2 cells has been acknowledged to be a significant contributor to the onset of asthma. Nevertheless, further research has indicated that the Th1/Th2 cell imbalance theory is unable to account for various experimental phenomena. In addition, the pathogenesis of asthma has progressed to include attention given to Th17 cells and Tregs.

## The main mechanism of eosinophilic asthma is the type 2 inflammatory response mediated by Th2 cells and ILC2s

Eosinophilic asthma is the most common type of asthma found in patients with asthma of varying severity and is now thought to be primarily associated with a type 2 inflammatory response mediated by Th2 cells and Type II innate lymphocytes (ILC2s) [14–16].

Foreign antigens enter the body and are captured by antigen-presenting cells (APCs), such as DCs. Here, they form MHC II-antigen-peptide complexes with the help of MHC class II molecules. T cell receptors (TCRs) on naïve CD4 + T cells (Th0) recognize these complexes. In the presence of signals generated by co-stimulatory molecules (CD80, CD86, OX40 and its ligands) and in the presence of IL-4, Th2 cells and follicular helper T (Tfh) cells differentiate. Consequently, they release type 2 cytokines, which trigger the maturation and recruitment of other immune cells involved in the allergic cascade. Additionally, these cytokines promote the accumulation of activated Th2 cells, eosinophils, ILC2s, DCs, macrophages and natural killer T cells (NKTs) in lung tissue. Frequent tissue damage triggers a reparative course that accelerates the deposition of connective tissue and the modification of airway configuration, resulting in thickening of the basement membrane and smooth muscle hypertrophy, recognized as airway remodeling [17, 18].

Allergen-specific Th2 cells, which produce IL-4, IL-5, IL-13 and even IL-9, have been found in the blood of individuals with allergic asthma [19]. The function of these cytokines in asthma has been extensively debated. Studies have indicated that IL-4 primarily originates from mast cells, NKT cells, γδT cells, Th2 cells, ILC2s and basophils. It interacts with B cells, induces IgE class switch recombination, B-cell maturation, and promotes the expression of adhesion molecules (intercellular adhesion molecule-1 [ICAM-1] and vascular cell adhesion molecule-1 [VCAM-1]) to facilitate eosinophil extravasation [20]. Both IL-4 and IL-13 bind to the IL-4Rα chain to initiate the corresponding response. This finding suggests that there may be partial overlap in their functions. IL-13 plays an important role in increased mucus production, goblet cell metaplasia and airway hyperresponsiveness. Furthermore, periostin gene expression in bronchial epithelial cells is upregulated by IL-13 and IL-4 [21]. Periostin acts on fibroblasts to promote airway remodeling, enhance mucus secretion and recruit eosinophils. IL-5 facilitates eosinophil development, activation, migration, proliferation and survival. Research conducted in mice has shown that IL-5 promotes eosinophil recruitment and activation, but its role in promoting AHR has been controversial [22]. The contribution of Th9 cells, a distinctive subset of T-cells that produce IL-9, to asthma pathogenesis remains debatable as there is a paucity of research studies covering this topic. When anti-IL-9 antibodies were administered, exposure to allergens in mouse models resulted in positive outcomes, such as decreased expression of pertinent cytokines, decreased lung eosinophil counts, and decreased AHR [23]. However, a study indicated that the forced expiratory volume in 1 s (FEV1) level, incidence of asthma exacerbation, and quality of life in patients failed to benefit from treatment with the humanized IL-9 neutralizing antibody MEDI-528, also known as enoclizumab [24]. These studies suggest a potential correlation between IL-9 and AHR, pulmonary eosinophilia, and mucus hypersecretion.

Tfh cells are a distinct subpopulation of CD4 + T cells that orchestrate paracrine signaling from B cells to stimulate plasma cell differentiation and IgE production. In a mouse model of house dust mite (HDM) sensitization, Tfh cells were shown to reside in the lungs draining mediastinal lymph nodes subsequent to the initial sensitization phase. These cells then differentiate into Th2 cells and migrate to the lungs to execute their functions following a second exposure to HDM allergens [25]. It is noteworthy that Tfh cells induce IgE production, even in patients where conventional Th2 cells are not present or have limited effector cell capacity [26].

**Fig. 1:**
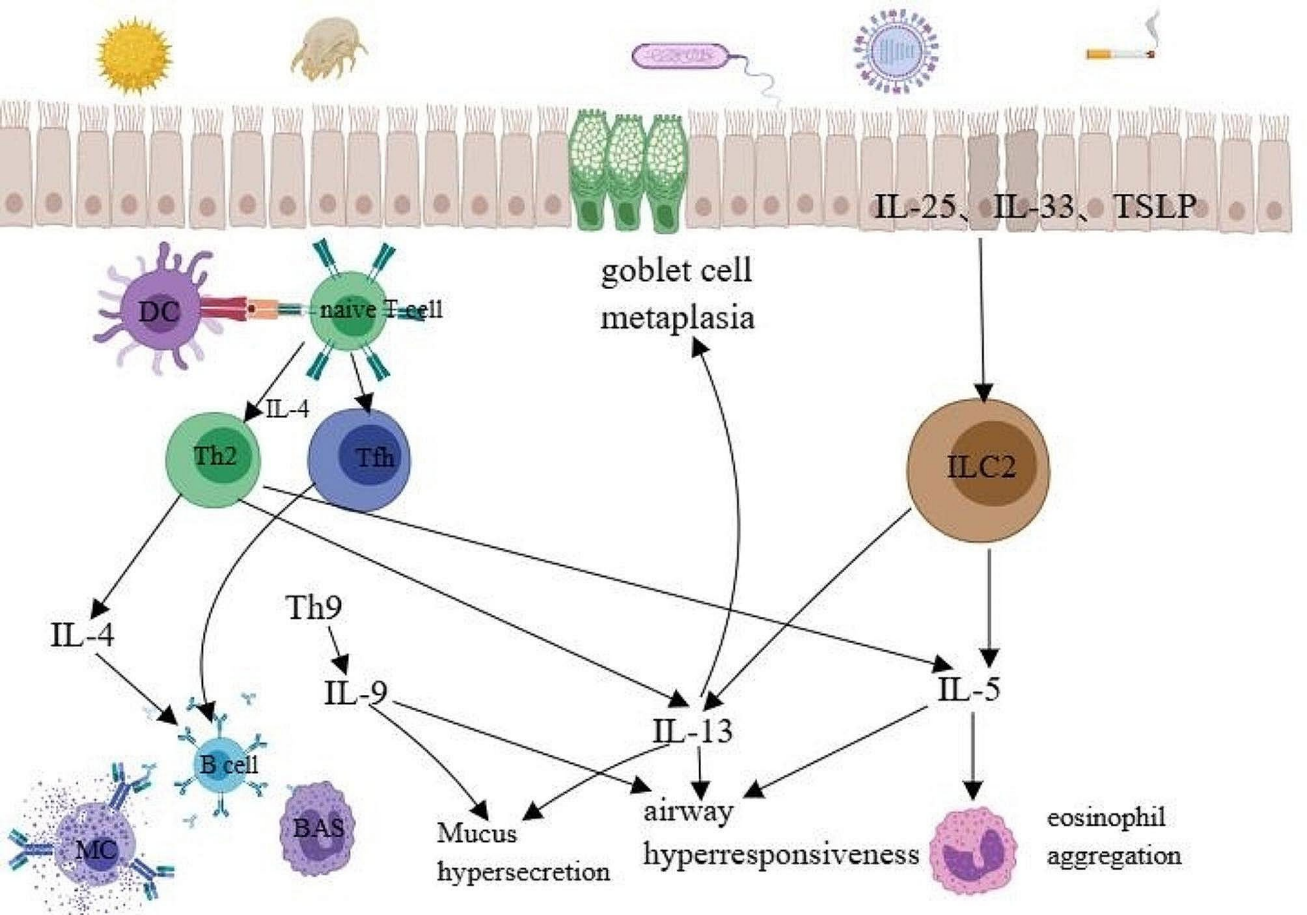
Involvement of Th2 and ILC2 cell-associated cytokines in the development of asthma

ILC2s represent a recently identified lymphocyte subset producing type 2 cytokines, particularly IL-5 and IL-13. Several studies have shown that ILC2s play an important role in the development of asthma. When airway epithelial cells are harmed by allergens, pollutants, or infections, pattern recognition receptors (PRRs) on the cells can identify pathogen-associated molecular patterns (PAMPs) present in inhaled bacteria and parasites, as well as damage-associated molecular patterns (DAMPs) produced by dead or injured cells. The activation of downstream signals and the release of cytokines, including IL-25, IL-33 and thymic stromal lymphopoietin (TSLP), by epithelial cells stimulate the differentiation and activation of ILC2s and other innate immune cells (such as macrophages, basophils, and eosinophils). This leads to the enhancement of the type 2 immune response [27–30][Fig. 1].

TSLP, IL-25, and IL-33 are regarded as primary molecules involved in type 2 inflammatory response and have a crucial role in the pathogenesis of asthma. IL-25 levels were found to be higher in individuals with eosinophilic asthma. Elevated IL-25 levels are associated with enhanced airway hyperresponsiveness, increased numbers of airway and blood eosinophils, elevated serum IgE levels, and subepithelial stromal deposits [31]. IL-25 acts directly on bronchial epithelial cells (BECs) and circulating fibroblasts (CFs) in autocrine and paracrine-dependent manners, respectively, promoting airway remodeling and fibrosis in patients with asthma [32]. IL-33 is elevated in the epithelial cells and bronchoalveolar lavage (BAL) fluid of individuals with asthma and is clearly correlated with the severity of the disease [33]. As a resident in lung tissue, ILC2s express significantly high levels of IL-33R. Activation of ERK1/2, p38 MAPK, AKT, JNK, and nuclear factor-κB (NF-κB) by IL-33 can cause chemotaxis of ILC2s, ultimately promoting the accumulation of immunological cells in the lung [34]. TSLP mRNA expression increases in the airway mucosa of asthmatic individuals and is positively correlated with disease severity [35]. Studies have shown that TSLP promotes ILC2 lifespan and glucocorticoid resistance [36]. Furthermore, although TSLP has traditionally been linked to type 2 inflammation, its expression in BAL fluid has been found to also have an association with neutrophil inflammation [37].

ILC2s not only mediate eosinophil aggregation, goblet cell metaplasia, and airway hyperresponsiveness through the production of IL-5, IL-9, and IL-13 but also may act as APCs to provide CD4 + T lymphocytes with MHCII molecules and OX40 ligands (OX40L). Activation of the ILC2s in the lung has been reported to induce key asthma features such as eosinophil aggregation and airway hyperresponsiveness in mouse models lacking T cells and B cells [17]. Therefore, we believe that the roles of Th2 cells and ILC2s in the pathogenesis of asthma are complementary.

Drugs that target type 2 cytokines and their receptors are now integral to asthma treatment. A humanized monoclonal IL-13 neutralizing antibody called lebrikizumab was found to reduce asthma exacerbations and improve lung function in patients with moderate-to-severe asthma in a clinical trial. Lebrikizumab also demonstrated good therapeutic effects in asthma patients with high levels of periosteal proteins [38]. Lebrikizumab treatment had no impact on tissue or blood eosinophil counts, which could account for its ineffectiveness in managing asthma exacerbations. In contrast, dupilumab, a monoclonal antibody that targets the IL-4R alpha chain common to both the IL-4 and IL-13 receptors, prevents transduction signals stimulated by IL-4 and IL-13, and has been utilized for asthma treatment. However, it did not have any significant impact on those with non-eosinophilic asthma. In eosinophilic asthma patients, the drug lessened the number of asthma attacks and glucocorticoid usage. Mepolizumab, a human IL-5 monoclonal antibody, significantly decreases blood eosinophil counts, decreases the incidence of acute exacerbations, and enhances the quality of life of patients with severe eosinophilic asthma [39, 40].

Clinical trials of drugs targeting type 2 cytokines and their receptors have yielded useful results, and a variety of drugs targeting key molecules of the type 2 inflammatory response are now in use, providing better options for the treatment of asthma, which in turn confirms the role of these cytokines in the pathogenesis of asthma. In addition to therapeutic methods focused on type 2 cytokines, which are the molecules that trigger type 2 inflammatory responses, investigations concerning epithelial-derived cytokines such as IL-25, IL-33, and TSLP have been conducted and have provided insight into novel drug development prospects [41, 42].

## The role of Th17 cells and Th1 cells in neutrophilic asthma

Some asthma patients do not exhibit tissue eosinophilia, but instead exhibit neutrophilia. This type of asthma may be associated with severe asthma and glucocorticoid-resistant asthma. The development of this part of asthma may be related to the immune response of Th17 cells and Th1 cells, and the exact mechanism still needs to be further investigated [43, 44].

The mechanism by which Th17 cells recruit neutrophils is not fully understood. IL-17A protein expression in sputum from asthmatics was found to be positively correlated with increased neutrophil count and airway hyperresponsiveness [45]. Overexpression of IL-17A in a mouse model resulted in the generation of a large number of peripheral neutrophils and enhanced granulopoiesis, suggesting that IL-17A significantly promoted neutrophil maturation, migration, and proliferation [46]. Increased airway neutrophil infiltration may result from the ability of IL-17A to increase the secretion of neutrophil chemokines, such as CXCL-8, and from the ability of airway epithelial cells, IL-1β, IL-6, and GM-CSF to proliferate. Reactive oxygen species (ROS), neutrophil elastase (NE), and matrix metalloproteinase 9 (MMP-9) are produced by neutrophils [47, 48]. MMP-9 stimulates DC migration and maturation, and NE causes mucus secretion by goblet cells. These processes negatively regulate tissue inhibitor of matrix metalloproteinases 1 (TIMP-1), which in turn promotes mucus production, bronchoconstriction, and tissue remodeling. Furthermore, neutrophil extracellular traps (NETs)—structures made of DNA, modified histones, and granule proteins such as NE and myeloperoxidase (MPO)—can be produced by activated neutrophils. These structures can disturb the integrity of bronchial epithelial cells, leading to the release of cytokines such as TSLP and IL-33, which can exacerbate the pathology of asthma [49]. Moreover, discoveries from a mouse model indicate that IL-17A intensifies smooth muscle contraction and worsens airway hyperreactivity by activating NF-κB, which results in an increase of RhoA and ROCK2 expression [50].

However, whether the cytokines IL-17F and IL-22 produced by Th17 cells are protective or pathogenic in asthma pathogenesis remains to be further investigated. IL-17F inhibits the activation of DCs, which results in a diminished Th2 response and decreased airway inflammation. These findings may provide a possible explanation for the increased allergic responses found in IL-17F-deficient mice in some studies [51]. However, it has been shown that IL-17F enhances airway smooth muscle contraction, migration and proliferation, thereby promoting asthmatic features such as airway hyperresponsiveness and airway remodeling. In a mouse model of sensitization to ovalbumin (OVA), IL-22 contributes significantly to the inflammation of airways triggered by allergen inhalation through eosinophils and neutrophils [52]. However, a different study found that introducing the IL-22 gene before systemic sensitization restrained antigen-stimulated CD4 + T cell expansion and the inflammation in the airways caused by eosinophils [53].

However, the function of Th17 cells in the pathogenesis of asthma remains controversial. While in models of OVA antigen sensitization, Th17 cells encourage neutrophil recruitment to the airways and contribute to the development of AHR, in other models, the Th17 response has a negative regulating effect on the Th2 response, thus halting AHR development. In clinical trials conducted on neutrophilic asthmatic patients, the administration of a CXCR2 antagonist to block CXCL8-mediated neutrophil recruitment did not affect any asthma outcomes. Therefore, the role of neutrophils in asthma remains uncertain, and it is unknown whether they are active participants or passive bystanders [54].

On the other hand, TNF-α and IFN-γ represent crucial cytokines that are discharged during the.

**Fig. 2:**
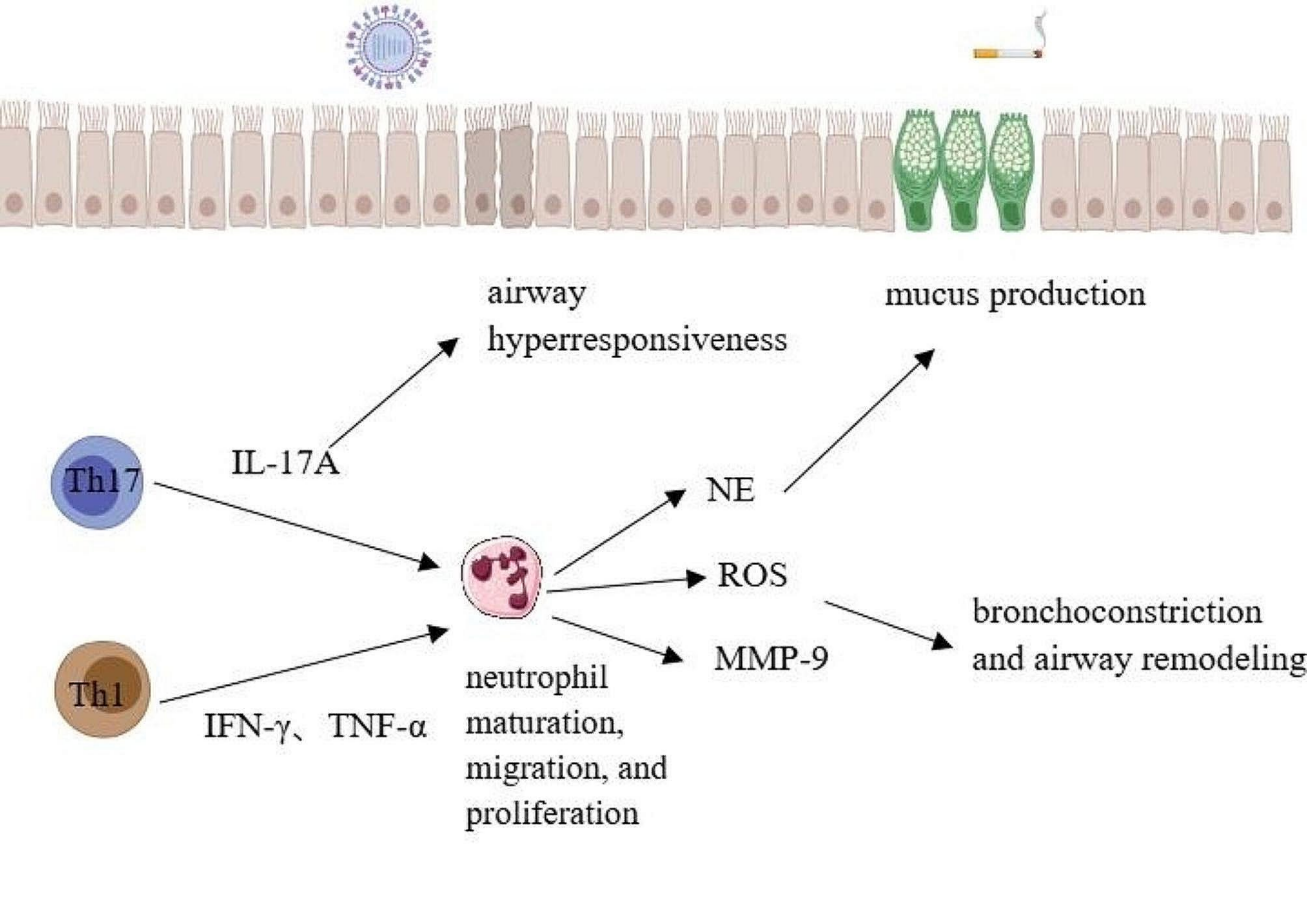
Th1 and Th17 promote neutrophilic inflammation

Th1 immune response and cause the release of pro-inflammatory mediators, airway hyperresponsiveness and airway remodeling [55]. Patients suffering from neutrophilic asthma exhibit elevated sputum TNF-α levels, and it is believed that TNF-α is involved in neutrophilic asthma by promoting concurrent neutrophil recruitment with the cytokine IL-17A. It has been demonstrated that IFN-γ + CD4 + T cells are predominant in the BAL fluid of children with severe asthma suffering from both eosinophilic and neutrophilic asthma [Fig. 2]. This study suggests that the Th1 immune response is related to severe asthma [56]. Furthermore, TNF-α and IFN-γ levels are increased in certain patients who are resistant to corticosteroid therapy. The investigation presents the possibility that interactions between TNF-α and IFN-γ play a role in corticosteroid resistance among asthma patients [57]. It has been shown that the Th1 cytokine IFN-γ promotes neutrophil recruitment in the presence of the cytokine IL-17 [58].

However, the exact potential of blocking IL-17A or its receptor for treating neutrophilic asthma remains unclear [59]. Whether and how neutrophilic inflammation can be a target for asthma treatment requires further in-depth study. In addition, studying the relationship between neutrophilic asthma and hormone resistance may be useful in the treatment of refractory asthma.

## The role of regulatory T cells (Tregs) in the development of asthma

Tregs are a specialized population of CD4 + T cells that act to suppress the immune response, thereby maintaining the body’s immune homeostasis and self-tolerance. Reduced numbers of Tregs have been observed in the blood and induced sputum of patients with asthma, and during acute asthma exacerbations, asthmatics have a greater degree of Tregs deficiency [60, 61]. In addition to their reduced numbers, the ability of Tregs to suppress effector T cells and their cytokines is significantly reduced in allergic individuals [62, 63]. In a mouse model, adoptive transfer of CD4 + CD25 + Tregs to mice prior to antigen challenge inhibited the development of allergic disease, and adoptive transfer of Tregs after disease onset attenuated established inflammation and airway remodeling [64, 65]. These studies support the idea that decreased numbers of Tregs and functional deficits contribute to asthma. Tregs can be divided into two main subpopulations: thymus-derived Tregs (tTregs) and peripherally-derived Tregs (pTregs). tTregs primarily recognize self-antigens, which is important for maintaining self-tolerance and preventing autoimmunity. However, pTregs are thought to be involved primarily in the tolerogenic response to foreign antigens and microbes [66, 67].

Tregs may mediate immunomodulatory functions through a variety of mechanisms, including the release of inhibitory cytokines (IL-10, TGF-β, and IL-35, etc.), the release of cytolytic molecules [granzymes (Gzm) A and B], and the regulation of the expression of specific transcription factors and receptors and so on. Tregs can modulate the activation of various cell types involved in allergic responses, such as eosinophils, neutrophils, mast cells, and B cells, through the mechanisms mentioned above [68].

IL-10, TGF-β, and IL-35 are considered the major factors involved in mediating the regulatory functions of Tregs [69].

IL-10 is a cytokine produced by various cell types and is implicated in the regulatory functions of Tregs. The transfer of allergen-specific CD4 + CD25 + Tregs into OVA-sensitized mice significantly reduced AHR and inflammatory cell recruitment, and IL-10 levels were elevated in the BAL fluid of the transferred mice. Anti-IL-10R treatment abolished the inhibition of AHR and lung eosinophil infiltration by CD4 + CD25 + regulatory cells when neutralizing antibodies against the IL-10 receptor were administered during the allergen challenge phase [64]. Tregs release of IL-10 obstructs the production of pro-inflammatory cytokines and restricts the antigen-presenting function of APCs. Furthermore, IL-10 regulates the function of diverse immune cells, including mast cells, Th2 cells, eosinophils, and DCs, which are implicated in allergic responses [70, 71].

Transforming growth factor-β (TGF-β) is a pleiotropic cytokine released by Tregs that is involved in the regulation of the immune response. On the one hand, it directly prevents the proliferation of T cells and B cells and inhibits the proliferation and function of macrophages by reducing the release of various cytokines. In addition, TGF-β is involved in the regulation of airway inflammation, tissue repair and other processes. However, the contribution of TGF-β to the immunosuppressive function of Tregs remains controversial. It has been reported that the lack of TGF-β in mice does not affect their immunosuppressive capacity [72]. On the other hand, TGF-β not only has anti-asthmatic effects in the pathogenesis of asthma, but also has pro-asthmatic effects. TGF-β is an important participant in airway remodeling. The current study shows a significant correlation between the number of epithelial or submucosal cells expressing TGF-β, the thickness of the basement membrane and the number of fibroblasts in asthma [73]. TGF-β induces an increase in the number of fibroblasts and airway smooth muscle proliferation, which in turn leads to extracellular matrix (ECM) deposition and ultimately exacerbates the process of airway remodeling [74].

IL-35 is a vital cytokine that Tregs release and directly affects effector cells. In patients with allergic asthma, the protein and mRNA levels of IL-35 are lower than those in healthy controls [75]. Additionally, it has been recognized that this cytokine is produced mainly by Tregs and has anti-inflammatory and immunosuppressive effects. Research has suggested that the absence of one of the two subunits diminishes the inhibitory ability of Tregs, affirming the function of IL-35 in immune system suppression in an inflammatory bowel disease (IBD) mouse model [76]. IL-10 and TGF-β are not essential for inhibiting IL-17-dependent AHR after airway sensitization; in particular, IL-35 performs as the primary cytokine for this purpose [77]. Therefore, these studies demonstrate the crucial involvement of IL-35 in the pathogenesis of asthma.

Stimulation by various cytokines results in the differentiation of naïve T cells into discrete subpopulations under the guidance of lineage-specific transcription factors, including T-bet (Th1 cells), GATA3 (Th2 cells), RORγt (Th17 cells) and FOXP3 (Tregs). The differentiation mechanisms induced by transcription factors are mutually exclusive, resulting in functional suppression between T-cell subsets. This phenomenon is especially noticeable between effector Th cells and Tregs, for instance, between Th2 cells and Tregs and between Th17 cells and Tregs. Imbalances in the immune response of these effector and regulatory cells have been linked to the development of asthma.

Both Th17 cells and Tregs require TGF-β for differentiation, but this process depends on the presence or absence of IL-6 [78]. It has been shown that the presence of IL-6 promotes the differentiation of primitive T cells to Th17 cells. However, in an environment lacking IL-6, primitive T cells differentiate into Tregs.

In addition, the Th2 cytokine IL-4 first activates the transcription factor STAT6, followed by the activation of GATA-3, which leads to the differentiation of primitive T cells into Th2 cells and promotes the expression of Th2 cell-related genes. TGF-β can bind to its receptor, activate Smad family transcription factors, promote FOXP3 transcription, and promote Treg-related gene expression. Moreover, GATA-3 can directly bind to the FOXP3 promoter and inhibit the expression of Treg-related genes, thus inhibiting the differentiation of primitive T cells into Tregs. FOXP3 also binds to GATA-3 and prevents it from regulating the expression of Th2 genes. Since GATA-3 and FOXP3 inhibit each other’s functional activity, the relative amounts of GATA-3 and FOXP3 may determine the fate of CD4 + T cells to differentiate into Th2 cells or Tregs [79].

The balance between the immune response of Th cells and the immunosuppression of Tregs is important for the maintenance of immune homeostasis. However, in recent years, it has been discovered that transcription factors produced by effector Th cells and their corresponding cytokines are involved not only in the immune responses of effector Th cells but also in the immunosuppression of Tregs to prevent excessive immune responses [80]. The treatment of asthma by targeting Tregs is currently immature. Rapamycin is a novel macrolide immunosuppressive drug that has been widely used for the prevention of clinical allograft rejection and some autoimmune diseases. Experiments in mouse models have shown that rapamycin promotes Tregs differentiation and induces organismal immune tolerance. Further studies on the mechanism of Tregs regulation may provide new ideas for clinical treatment [81].

## The role of additional factors in the development of asthma

### Eosinophils

Eosinophils play a pivotal role in asthma, as their accumulation in lung tissue is stimulated by IL-5, thereby fostering eosinophil formation in the bone marrow and attracting eosinophils to the lung mucosa and mesenchyme via eosinophil chemokines (e.g., eotaxins). Upon stimulation, eosinophils release large amounts of inflammatory mediators, including cytokines (such as IL-13 and IL-5), chemokines, granule mediators, and cysteinyl leukotrienes (cysLTs). Eosinophils produce cytotoxic proteins, including major basic protein (MBP), eosinophil peroxidase (EPX), eosinophil cationic protein (ECP) and eosinophil-derived neurotoxin (EDN), which are held in intracellular granules. These proteins contribute to the remodeling of airways, increased airway responsiveness and mucus generation [82]. Eosinophils also release extracellular deposits of genetic material that interweave to form a network known as eosinophil extracellular traps (EETs). EETs have an inherent function in fostering eosinophil degranulation and prompting the production of IL-6 and IL-8 by epithelial cells. It not only has an important function in immunity against extracellular pathogens but also contributes to the pathogenesis of asthma [83]. Increased production of EETs can increase mucus viscosity, leading to airway obstruction. In patients with severe asthma, the number of peripheral eosinophils producing EETs is elevated, and these cells stimulate lung epithelial cells to generate IL-33 and TSLP [84]. In addition, eosinophils can impact the performance of other immune cells through cytokines and chemokines. For instance, they can promote Th2 cell differentiation by secreting the Th2-associated cytokine IL-4.

### Mast cells and basophils

Mast cells and basophils play comparable roles in asthma pathogenesis and participate in the organism’s reaction to allergens. During the sensitization phase, IgE binds to the IgE high-affinity receptor FcεRI, which both mast cells and basophils express. Following re-exposure to allergens, cross-linking between antigenic peptides and allergen-specific IgE molecules provokes the degranulation of mast cells and basophils. Pre-synthesized mediators found in mast cells are released, consisting of histamine and proteases. Additionally, newly formed mediators, including cytokines, chemokines, prostaglandins, and leukotrienes, are unleashed, serving to further stimulate the inflammatory response characteristic of asthma. The release of mast cell mediators induces a local inflammatory response, accompanied by smooth muscle contraction, amplified capillary dilation and permeability, and heightened glandular secretions. These responses augment the infiltration of lymphocytes and eosinophils into the inflamed site [85]. Mast cell degranulation is directly related to asthma severity. The severity of asthma is closely linked to the degranulation of mast cells. Mast cell-produced chymotrypsin is a protease that undermines the integrity of the epithelial barrier, worsening EC destruction [86]. In the airway, the release of LTC4, PGD2, IL-4, and IL-13 from mast cells promotes the secretion of large amounts of mucus by goblet cells [87]. In addition, mast cells and eosinophils promote type 2 cytokine production. Mast cell localization within the airway smooth muscle is a crucial characteristic in the development of asthma. It contributes to hypertrophy and hyperplasia of smooth muscle and facilitates the establishment of AHR [88]. Basophils enhance B-cell proliferation, category-switching reorganization, plasma cell differentiation and maturation, and immunoglobulin production [89].

### IgE

Elevated serum IgE levels are a crucial indicator for patient assessment and strongly correlate with asthma development [90]. Research suggests that heightened IgE levels are associated with airway hyperresponsiveness, bronchial wall thickening, and severe asthma [91, 92]. IgE has been shown to directly affect eosinophil function by activating and releasing eosinophil peroxidase, expressing integrins, and releasing TNF-α. Furthermore, IgE stimulates airway smooth muscle directly, resulting in the production of cytokines (IL-4, IL-5, IL-13, TNF-α and TSLP) and chemokines (CCL5, CCL11, CXCL8 and CXCL10). These substances are responsible for the contraction and proliferation of airway smooth muscle, eventually leading to airway remodeling [93].

Omalizumab is currently the most advanced anti-IgE monoclonal antibody of human origin. It functions by intercepting the allergic cascade process via the prevention of the binding of IgE to the FcεRI receptor on mast cells, basophils, and other inflammatory cells. Several clinical trials conducted in both children and adults have reported positive outcomes. Therefore, omalizumab is extensively used in clinical practice. Omalizumab has favorable effects, including decreasing sputum eosinophil counts, reducing airway hyperresponsiveness, and improving symptoms. Moreover, the use of omalizumab has led to a decrease in the frequency of acute exacerbations and hospitalizations, along with a reduction in steroid use and an improvement in asthma control [94].

## Discussion

The development of asthma is driven by the participation of various immune cells and molecules. Despite the increased options available for investigating disease pathogenesis in current disease models, our comprehension of its pathogenesis remains inadequate. Molecular biology and immunology advancements have given way to selective blocking agents that offer an original perspective for treating asthma patients. Inhibiting crucial molecules involved in asthma pathogenesis has significantly contributed to advancements in asthma treatment. Studying the cellular and molecular mechanisms involved in asthma-related inflammation might unveil novel therapeutic targets that could enhance the efficacy of asthma drugs. Additionally, these findings could inform the quest for more suitable biomarkers.

## Data Availability

No datasets were generated or analysed during the current study.
